# 2,2,3,3-Tetra­fluoro­butane-1,4-diol

**DOI:** 10.1107/S1600536808040555

**Published:** 2008-12-06

**Authors:** Moritz M. Reichvilser, Felix W. Roessner, Peter Klüfers

**Affiliations:** a Ludwig-Maximilians-Universität, Department Chemie und Biochemie, Butenandtstrasse 5–13 (Haus D), 81377 München, Germany

## Abstract

In the title compound, C_4_H_6_F_4_O_2_, a partially fluorinated aliphatic diol, cooperative O—H⋯O hydrogen bonds form *R*_2_^2^(14) rings, which are connected into infinite layers parallel to the (100) plane by *C*(7) chains. A C—H⋯F link is also seen.

## Related literature

For crystal structures containing 2,2,3,3-tetra­fluoro­butane-1,4-di­oxy units, see: Elias *et al.* (1994 ▶); Beşli *et al.* (2004 ▶, 2005 ▶, 2006 ▶). For details on graph-set analysis of hydrogen-bond networks, see: Bernstein *et al.* (1995 ▶); Etter *et al.* (1990 ▶).
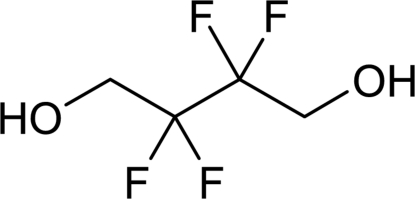


## Experimental

### 

#### Crystal data

C_4_H_6_F_4_O_2_
*M* *_r_* = 162.09
Monoclinic, 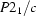

*a* = 5.4392 (2) Å
*b* = 8.6935 (3) Å
*c* = 12.4123 (4) Å
β = 99.768 (2)°
*V* = 578.42 (3) Å^3^
*Z* = 4
Mo *K*α radiation
μ = 0.22 mm^−1^
*T* = 200 (2) K
0.18 × 0.08 × 0.06 mm

#### Data collection

Nonius KappaCCD area-detector diffractometer
Absorption correction: none
2531 measured reflections
1313 independent reflections
1133 reflections with *I* > 2σ(*I*)
*R*_int_ = 0.020

#### Refinement

*R*[*F* ^2^ > 2σ(*F* ^2^)] = 0.031
*wR*(*F* ^2^) = 0.085
*S* = 1.04
1313 reflections
97 parameters
H atoms treated by a mixture of independent and constrained refinement
Δρ_max_ = 0.38 e Å^−3^
Δρ_min_ = −0.25 e Å^−3^

### 

Data collection: *COLLECT* (Hooft, 2004 ▶); cell refinement: *SCALEPACK* (Otwinowski & Minor, 1997 ▶); data reduction: *SCALEPACK* and *DENZO* (Otwinowski & Minor, 1997 ▶); program(s) used to solve structure: *SHELXS97* (Sheldrick, 2008 ▶); program(s) used to refine structure: *SHELXL97* (Sheldrick, 2008 ▶); molecular graphics: *ORTEP-3 for Windows* (Farrugia, 1997 ▶); software used to prepare material for publication: *SHELXL97* and *PLATON* (Spek, 2003 ▶).

## Supplementary Material

Crystal structure: contains datablocks I, global. DOI: 10.1107/S1600536808040555/zl2164sup1.cif

Structure factors: contains datablocks I. DOI: 10.1107/S1600536808040555/zl2164Isup2.hkl

Additional supplementary materials:  crystallographic information; 3D view; checkCIF report

## Figures and Tables

**Table 1:** Hydrogen-bond geometry (Å, °)

*D*—H⋯*A*	*D*—H	H⋯*A*	*D*⋯*A*	*D*—H⋯*A*
O1—H1⋯O2^i^	0.80 (2)	2.001 (19)	2.7972 (14)	171.9 (18)
O2—H2⋯O1^ii^	0.845 (17)	1.940 (17)	2.7608 (14)	163.6 (17)
C1—H1*B*⋯F3^i^	0.99	2.44	3.2343 (15)	137

Symmetry codes: (i) 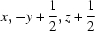; (ii) 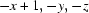.

**Fig. 1.**
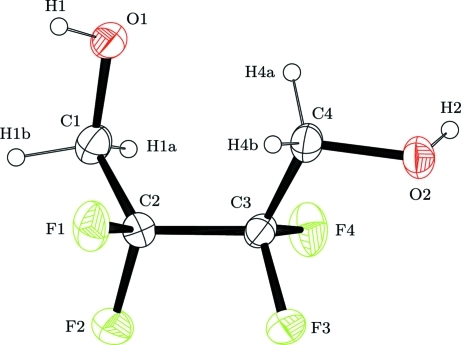
The molecular structure of the title compound, with atom labels and anisotropic displacement ellipsoids (drawn at 50% probability level) for non-H atoms.

**Fig. 2.**
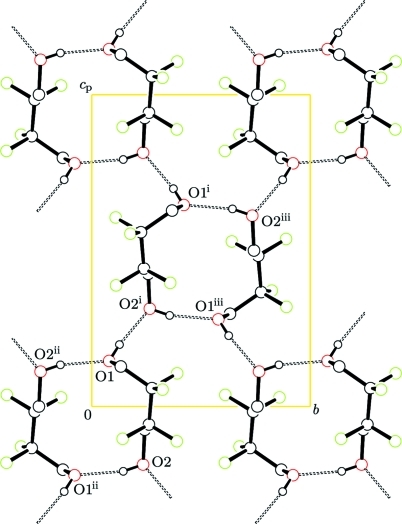
Hydrogen bonding in (I). [Symmetry codes: (i) x, 1/2-y, 1/2+z; (ii) 1-x, -y, -z; (iii) 1-x, 1/2+y, 1/2-z.]

**Fig. 3.**
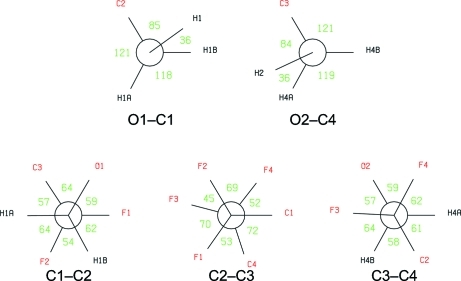
Newman projections along the bonds of the O1—C1—C2—C3—C4—O2 chain.

## supplementary crystallographic information

### Comment

The asymmetric unit of the title compound contains one complete molecule, which is shown in Figure 1.

The molecular packing is dominated by two O—H···O hydrogen bonds. According to graph set theory [Bernstein *et al.* (1995), Etter *et al.* (1990)] the descriptors *C*(7) and *R*_2_^2^(14) can be assigned. Together with one C—H···F hydrogen bond [motif *C*(5)] the first-level (unitary) graph set *N*_1_ = *C*(5)*C*(7)*R*_2_^2^(14) is obtained.

Figure 2 shows a cutout of the layers parallel to the (100) plane which are generated by the O—H···O hydrogen bond framework. The C—H···F bonds which are located within these layers are omitted for clarity.

Due to packing effects and the specific hydrogen bonding interactions the O1—C1—C2—C3—C4—O2 chain adopts a somewhat unusual conformation. The substituents at the C2—C3 fragment are staggered with the CH_2_OH moieties being gauche to each other. For Newman projections see Fig. 3.

### Experimental

The title compound was obtained from Acros Organics. A single-crystal suitable for X-ray diffraction was isolated from the supplied material.

### Refinement

All H atoms were found in difference maps. C-bonded H atoms were positioned geometrically (C—H = 0.99 Å) and treated as riding on their parent atoms [*U*_iso_(H) = 1.2*U*_eq_(C)]. Coordinates of O-bonded H atoms and O—H distances were refined freely [*U*_iso_(H) = 1.5*U*_eq_(O)].

### Crystal data

**Table:**

C_4_H_6_F_4_O_2_	*F*(000) = 328
*M**_r_* = 162.09	*D*_x_ = 1.861 (1) Mg m^−^^3^
Monoclinic, *P*2_1_/*c*	Melting point = 355.3–356.3 K
Hall symbol: -P 2ybc	Mo *K*α radiation, λ = 0.71073 Å
*a* = 5.4392 (2) Å	Cell parameters from 7007 reflections
*b* = 8.6935 (3) Å	θ = 3.1–27.5°
*c* = 12.4123 (4) Å	µ = 0.22 mm^−^^1^
β = 99.768 (2)°	*T* = 200 K
*V* = 578.42 (3) Å^3^	Block, colourless
*Z* = 4	0.18 × 0.08 × 0.06 mm

### Data collection

**Table:**

Nonius KappaCCD area-detector diffractometer	1133 reflections with *I* > 2σ(*I*)
Radiation source: rotating anode	*R*_int_ = 0.020
MONTEL, graded multilayered X-ray optics	θ_max_ = 27.5°, θ_min_ = 3.3°
Detector resolution: 9 pixels mm^-1^	*h* = −7→7
φ and ω scans	*k* = −11→11
2531 measured reflections	*l* = −16→16
1313 independent reflections	

### Refinement

**Table:**

Refinement on *F*^2^	Primary atom site location: structure-invariant direct methods
Least-squares matrix: full	Secondary atom site location: difference Fourier map
*R*[*F*^2^ > 2σ(*F*^2^)] = 0.031	Hydrogen site location: difference Fourier map
*wR*(*F*^2^) = 0.085	H atoms treated by a mixture of independent and constrained refinement
*S* = 1.04	*w* = 1/[σ^2^(*F*_o_^2^) + (0.0398*P*)^2^ + 0.2156*P*] where *P* = (*F*_o_^2^ + 2*F*_c_^2^)/3
1313 reflections	(Δ/σ)_max_ < 0.001
97 parameters	Δρ_max_ = 0.38 e Å^−^^3^
0 restraints	Δρ_min_ = −0.25 e Å^−^^3^

### Special details

**Table:**

Geometry. All e.s.d.'s (except the e.s.d. in the dihedral angle between two l.s. planes) are estimated using the full covariance matrix. The cell e.s.d.'s are taken into account individually in the estimation of e.s.d.'s in distances, angles and torsion angles; correlations between e.s.d.'s in cell parameters are only used when they are defined by crystal symmetry. An approximate (isotropic) treatment of cell e.s.d.'s is used for estimating e.s.d.'s involving l.s. planes.
Refinement. Refinement of *F*^2^ against ALL reflections. The weighted *R*-factor *wR* and goodness of fit *S* are based on *F*^2^, conventional *R*-factors *R* are based on *F*, with *F* set to zero for negative *F*^2^. The threshold expression of *F*^2^ > 2*σ*(*F*^2^) is used only for calculating *R*-factors(gt) *etc*. and is not relevant to the choice of reflections for refinement. *R*-factors based on *F*^2^ are statistically about twice as large as those based on *F*, and *R*-factors based on all data will be even larger.

### Fractional atomic coordinates and isotropic or equivalent isotropic displacement parameters (Å^2^)

**Table:**

	*x*	*y*	*z*	*U*_iso_*/*U*_eq_	
F1	0.70269 (17)	0.39078 (9)	0.15846 (6)	0.0318 (2)	
F2	1.07701 (15)	0.32375 (11)	0.13418 (7)	0.0343 (2)	
F3	0.81808 (17)	0.38903 (11)	−0.05076 (6)	0.0349 (2)	
F4	0.86586 (15)	0.14104 (11)	−0.03922 (6)	0.0328 (2)	
O1	0.58505 (19)	0.07917 (11)	0.18952 (8)	0.0256 (2)	
H1	0.528 (4)	0.125 (2)	0.2357 (16)	0.038*	
O2	0.41094 (18)	0.23464 (12)	−0.15572 (7)	0.0253 (2)	
H2	0.431 (3)	0.143 (2)	−0.1751 (15)	0.038*	
C1	0.8308 (3)	0.13254 (16)	0.19011 (10)	0.0246 (3)	
H1A	0.9293	0.0505	0.1621	0.030*	
H1B	0.9099	0.1556	0.2662	0.030*	
C2	0.8355 (2)	0.27521 (15)	0.12090 (10)	0.0216 (3)	
C3	0.7415 (2)	0.26145 (14)	−0.00258 (10)	0.0202 (3)	
C4	0.4637 (2)	0.24221 (16)	−0.04003 (10)	0.0227 (3)	
H4A	0.4061	0.1468	−0.0086	0.027*	
H4B	0.3737	0.3302	−0.0142	0.027*	

### Atomic displacement parameters (Å^2^)

**Table:**

	*U*^11^	*U*^22^	*U*^33^	*U*^12^	*U*^13^	*U*^23^
F1	0.0491 (5)	0.0227 (4)	0.0240 (4)	0.0031 (4)	0.0075 (4)	−0.0057 (3)
F2	0.0285 (4)	0.0452 (5)	0.0273 (4)	−0.0150 (4)	−0.0012 (3)	0.0044 (4)
F3	0.0445 (5)	0.0363 (5)	0.0227 (4)	−0.0187 (4)	0.0026 (3)	0.0077 (3)
F4	0.0305 (5)	0.0407 (5)	0.0275 (4)	0.0102 (4)	0.0059 (3)	−0.0101 (4)
O1	0.0327 (5)	0.0239 (5)	0.0217 (5)	−0.0053 (4)	0.0091 (4)	−0.0022 (4)
O2	0.0325 (5)	0.0252 (5)	0.0169 (4)	−0.0018 (4)	0.0000 (4)	−0.0003 (4)
C1	0.0278 (7)	0.0261 (7)	0.0203 (6)	0.0001 (5)	0.0051 (5)	0.0028 (5)
C2	0.0226 (6)	0.0231 (6)	0.0189 (6)	−0.0036 (5)	0.0035 (5)	−0.0021 (5)
C3	0.0252 (6)	0.0193 (6)	0.0171 (6)	−0.0024 (5)	0.0068 (5)	−0.0004 (5)
C4	0.0238 (6)	0.0274 (7)	0.0165 (6)	−0.0005 (5)	0.0024 (5)	0.0001 (5)

### Geometric parameters (Å, °)

**Table:**

F1—C2	1.3651 (15)	C1—C2	1.5114 (18)
F2—C2	1.3626 (15)	C1—H1A	0.9900
F3—C3	1.3587 (14)	C1—H1B	0.9900
F4—C3	1.3656 (14)	C2—C3	1.5360 (17)
O1—C1	1.4135 (17)	C3—C4	1.5128 (17)
O1—H1	0.80 (2)	C4—H4A	0.9900
O2—C4	1.4173 (15)	C4—H4B	0.9900
O2—H2	0.84 (2)		
			
C1—O1—H1	108.0 (14)	C1—C2—C3	117.92 (11)
C4—O2—H2	108.6 (13)	F3—C3—F4	105.84 (9)
O1—C1—C2	111.96 (11)	F3—C3—C4	108.66 (10)
O1—C1—H1A	109.2	F4—C3—C4	109.78 (10)
C2—C1—H1A	109.2	F3—C3—C2	107.49 (10)
O1—C1—H1B	109.2	F4—C3—C2	106.97 (10)
C2—C1—H1B	109.2	C4—C3—C2	117.48 (10)
H1A—C1—H1B	107.9	O2—C4—C3	109.65 (10)
F2—C2—F1	106.61 (10)	O2—C4—H4A	109.7
F2—C2—C1	107.13 (10)	C3—C4—H4A	109.7
F1—C2—C1	110.41 (10)	O2—C4—H4B	109.7
F2—C2—C3	107.21 (9)	C3—C4—H4B	109.7
F1—C2—C3	106.98 (10)	H4A—C4—H4B	108.2
			
O1—C1—C2—F2	175.17 (10)	C1—C2—C3—F4	−52.09 (14)
O1—C1—C2—F1	59.45 (14)	F2—C2—C3—C4	−167.32 (11)
O1—C1—C2—C3	−63.91 (14)	F1—C2—C3—C4	−53.27 (14)
F2—C2—C3—F3	−44.50 (13)	C1—C2—C3—C4	71.80 (15)
F1—C2—C3—F3	69.55 (12)	F3—C3—C4—O2	56.60 (13)
C1—C2—C3—F3	−165.38 (11)	F4—C3—C4—O2	−58.71 (13)
F2—C2—C3—F4	68.79 (12)	C2—C3—C4—O2	178.82 (11)
F1—C2—C3—F4	−177.16 (9)		

### Hydrogen-bond geometry (Å, °)

**Table:**

*D*—H···*A*	*D*—H	H···*A*	*D*···*A*	*D*—H···*A*
O1—H1···O2^i^	0.80 (2)	2.001 (19)	2.7972 (14)	171.9 (18)
O2—H2···O1^ii^	0.845 (17)	1.940 (17)	2.7608 (14)	163.6 (17)
C1—H1B···F3^i^	0.99	2.44	3.2343 (15)	137

Symmetry codes: (i) *x*, −*y*+1/2, *z*+1/2; (ii) −*x*+1, −*y*, −*z*.
